# Current News

**Published:** 1891-10

**Authors:** 


### Current News.

### THE OHIO STATE DENTAL SOCIETY.

The Ohio State Dental Society holds its next annual meeting in the city of Columbus, on Tuesday, Wednesday, and Thursday, December 1, 2, and 3, 1891.

No pains will be spared to make this, in every way, an attractive meeting, and we extend a cordial invitation to members of the profession throughout the country to be present. Ample accommodations and liberal inducements will be given for the exhibition and demonstration of new and useful appliances, instruments, etc.

The geographical situation of Columbus and its extensive railroad connections make it easily accessible from all directions. Arrangements have been made with the Central Traffic Association for reduced railroad rates on the certificate plan.

E. G. Betty, *President*, Cincinnati.
Otto Arnold, *Secretary*, Columbus.

### THE FIFTH, SIXTH, SEVENTH, AND EIGHTH DISTRICT DENTAL SOCIETIES OF NEW YORK STATE.

The Fifth, Sixth, Seventh, and Eighth District Dental Societies of New York State will hold their Sixth Annual Joint Convention at the Iroquois Hotel, Buffalo, October 27, 28, 29. Of all the dental meetings held in this country, none are more profitable or more largely attended than these conventions, and the one this year promises to surpass all others in points of interest and attractiveness. Prominent men from this and adjoining States, and Canada, have already signified their intention to be present. An unusually interesting programme of essays and clinics is being prepared; there will also be a special collection of abnormal and pathological specimens relating to dentistry, and a cordial invitation is extended to all dentists to attend the meeting, and to contribute to the collection.

Special rates upon railroads will be secured, and all arrangements for the comfort and convenience of those attending will be made.

For any information regarding the meeting, address the chairman of the committee,

Charles S. Butler, Buffalo, N. Y.

### POST-GRADUATE ASSOCIATION OF THE UNITED STATES.

The annual meeting of the Post-Graduate Dental Association of the United States was held at the Leland Hotel, Chicago, June 24, Dr. Cushing, president, in the chair.

The order of the day was reports of officers, transaction of routine business, election of officers, and interesting discussions in regard to the future work of the society.

Officers elected for the ensuing year are as follows: President, Dr. R. B. Tuller, Chicago. Vice-Presidents, Dr. Levi S. Keagle, Vinton, Ia.; Dr. A. P. Nicholson, Edgerton, Wis., and Dr. M. R. Julian, Lafayette, Ind. Secretary and Treasurer, Dr. L. S. Tenney, Chicago.

This organization has just completed the second year of its existence and seems to have struck a popular chord in the profession, as its rapidly increasing membership would indicate.

As is generally well known, the object of the society is mainly to establish a systematic course of home study, and measures are now on foot to begin this work during the year.

Those desiring further information should address the Secretary, Dr. L. S. Tenney, 96 State Street, Chicago, Ill.

---

### NEW HAMPSHIRE DENTAL SOCIETY.

At the last annual meeting of the New Hampshire Dental Society, June 16, 1891, the following officers were elected: President, W. R. Blackston, Manchester; Vice-President, C. P. Webster, Franklin Falls; Treasurer, G. A. Young, Concord; Librarian, G. A. Bowers, Nashua.

*Executive Council.*—J. H. French, Penacook; E. C. Blaisdell, Portsmouth; E. B. Davis, Concord.

The Society will hold a special meeting at the Manchester House, Manchester, N. H., on September 29, 30, and October 1, 1891. All members of the profession are cordially invited to attend. Efforts are being made to make this the largest and most profitable meeting of dentists ever held in the State. Come and make it a success.

B. C. Russell,
*Secretary.*

Keene, N. H.

